# Clinical outcomes with bevacizumab-containing and non-bevacizumab–containing regimens in patients with recurrent glioblastoma from US community practices

**DOI:** 10.1007/s11060-015-1752-y

**Published:** 2015-03-15

**Authors:** Clara Chen, Arliene Ravelo, Elaine Yu, Rahul Dhanda, Ian Schnadig

**Affiliations:** 1 Department of Outcomes Research, McKesson Specialty Health and The US Oncology Network, The Woodlands, TX USA; 2 Genentech, Inc., Health Outcomes, South San Francisco, CA USA; 3 Department of Pharmacy and Therapeutics, Compass Oncology and The US Oncology Network, 19260 SW 65th Avenue, Suite 435, Tualatin, OR 97062 USA

**Keywords:** Bevacizumab, Recurrent glioblastoma, Community practice, Retrospective analysis

## Abstract

This analysis evaluated the efficacy and safety of bevacizumab as monotherapy and with irinotecan for recurrent glioblastoma in community-based practices. Adult patients with bevacizumab-naive, recurrent glioblastoma initiating second-line treatment (July 2006–June 2010) were identified using McKesson Specialty Health/US Oncology Network health records. Overall (OS) and progression-free survival (PFS) estimates were analyzed through July 2011 and compared for bevacizumab and non-bevacizumab regimens using the log-rank test. An adjusted Cox proportional hazards model assessed the effects of patient and treatment characteristics on outcomes. The analysis identified 159 patients initiating second-line treatment with a bevacizumab-monotherapy (*n* = 57), bevacizumab-combination (*n* = 79), or non-bevacizumab (*n* = 23) regimen. Patient characteristics were generally similar across groups. In the Cox analyses, OS (hazard ratio [HR] 0.51 [95 % confidence interval (CI) 0.31–0.82]; univariate medians: 8.86 vs. 5.19 months) was significantly longer with bevacizumab-containing regimens. Median PFS was longer with bevacizumab-containing regimens, but did not reach statistical significance (HR 0.64 [95 % CI 0.38–1.09]; univariate medians: 7.00 vs. 4.00 months). Analyses showed that each bevacizumab treatment group relative to non-bevacizumab had a reduced risk of death (bevacizumab-monotherapy regimen: HR 0.56 [95 % CI 0.31–1.03] and bevacizumab-combination regimen: HR 0.34 [95 % CI 0.21–0.68]). Patients receiving the bevacizumab-combination regimen trended toward longer OS and PFS than those receiving the bevacizumab-monotherapy regimen. Rates of bevacizumab-related toxicities were consistent with clinical trial reports.

**Electronic supplementary material:**

The online version of this article (doi:10.1007/s11060-015-1752-y) contains supplementary material, which is available to authorized users.

## Introduction

Glioblastoma, a high-grade malignant glioma, accounts for the majority of all primary brain tumor diagnoses in adults [1, 2]. Despite advances, prognosis remains poor, with a 14.6-month median overall survival (OS) in patients treated with the current frontline standard of care of surgical resection followed by fractionated radiotherapy and temozolomide [3, 4]. Recurrence is inevitable, and no universally accepted standard treatment at progression has been established in patients with unresectable disease [5].

Select patients with recurrent glioblastoma may be treated with additional surgery, radiotherapy, and/or second-line chemotherapy, including temozolomide, nitrosourea, cyclophosphamide, and platinum-based regimens [5]. Second-line chemotherapy confers a modest benefit, and has been associated with 6-month progression-free survival (PFS) rates of up to 15 % and a median OS approaching 6 months in patients with recurrent glioblastoma in phase 2 trials [6, 7].

Glioblastoma is among the most highly vascularized tumors, and is particularly suited to targeted antiangiogenic therapy [8]. In 2009, bevacizumab, a humanized monoclonal antibody against vascular endothelial factor (VEGF-A), was approved as a single-agent for patients with recurrent glioblastoma on the basis of phase 2 studies of bevacizumab both alone and in combination with irinotecan which demonstrated improvements in response rate and 6-month PFS relative to historic controls [9, 10]. A number of additional phase 2 studies, as well as retrospective community-practice-based studies have investigated bevacizumab in combination with other agents for recurrent, bevacizumab-naive glioblastoma, without signaling improved clinical efficacy over bevacizumab alone [11–23]. However, none of these community-based observational studies provided comparative outcomes data on bevacizumab-containing versus non-bevacizumab-containing regimens. Similarly, no randomized trials, to date, have been conducted in the recurrent setting to compare bevacizumab treatment to a control group receiving treatment other than bevacizumab or to alternate bevacizumab regimens, although one such study is currently enrolling patients [24].

In the current study, we describe differences in treatment characteristics and their association with efficacy and safety outcomes for bevacizumab monotherapy, bevacizumab-combination therapy, and non-bevacizumab–containing therapy in a large community-practice cohort of patients with bevacizumab-naive glioblastoma at first recurrence.

## Patients and methods

### Data source

In this retrospective cohort study, demographic, clinical, and treatment data were abstracted from the McKesson Specialty Health/US Oncology Network iKnowMed (iKM) electronic health record (EHR) database. iKM is an oncology-specific EHR system that captures outpatient practice encounter history from 900 community-based oncology providers across US Oncology Network practices or clinics in 39 states. For the timeframe of this study, iKM was active in approximately 82 % of the network, capturing data on outpatient medical oncology care for patients treated across 20 states. The study was conducted following approval by the institutional review boards of McKesson Specialty Health and US Oncology Network.

Baseline patient and clinical characteristics included age, sex, body mass index (BMI), practice region, payer status, blood pressure, and Karnofsky Performance Status (KPS). Vital status information was supplemented with data from the Social Security Death Index (SSDI). Additional data, including treatment, concomitant medication use, line of therapy, disease progression, adverse events, corticosteroid use during second-line therapy, and time since first surgery were captured through electronic chart reviews.

### Patients

The study included patients with recurrent histologically confirmed glioblastoma who initiated treatment following first recurrence, or second-line treatment, during the 4-year period between July 1, 2006, and June 30, 2010, with a minimum of 1-year of follow-up time with data cutoff at June 30, 2011. The analysis population met the following inclusion criteria: (1) confirmed diagnosis of glioblastoma, (2) receipt of care at any McKesson Specialty Health/US Oncology Network site using the full iKM EHR system before the start of second-line chemotherapy for glioblastoma, (3) age ≥18 years at diagnosis, and (4) at least two patient visits to practices during the study period. Patients were excluded if they were diagnosed with or treated for a primary cancer other than glioblastoma during the study period or were enrolled in a randomized clinical trial. Patients were divided into three groups on the basis of the composition of second-line therapy after the failure of a first-line regimen that did not include bevacizumab: bevacizumab monotherapy, bevacizumab therapy in combination with other treatments (bevacizumab combination), or non-bevacizumab–containing therapy (non-bevacizumab). There was no minimum duration of any component of second-line therapy required.

### Statistical analysis

Descriptive statistics with 95 % confidence intervals (CIs) were used to summarize patient and disease characteristics; characteristics were compared between groups using the Chi squared or exact tests for categorical variables and the F-test for continuous variables. OS was measured as the time from the initiation of second-line therapy to death or loss to follow-up. Patients alive at the end of follow-up (June 30, 2011) were censored for OS analyses. PFS was measured as the time from the initiation of second-line therapy to disease progression or death. The date of disease progression after the initiation of second-line therapy was abstracted from patients’ charts and was based on the physician’s notes of progressive disease and/or escalation in line of therapy. Disease progression was determined by a change in the line of therapy as documented in the iKM. For all patients on second-line therapy, chart review was performed and the line of therapy with initiation date (index date) was identified and maintained.

Estimates of PFS and OS with related 95 % CIs were calculated using Kaplan–Meier methods. The log-rank test was applied to compare survival time between treatment groups. A Cox proportional hazards model was developed to assess independent effects of patient and treatment characteristics on the survival outcomes of interest while controlling for the following potential confounding variables: age (< 60 years or ≥ 60 years), sex, BMI (<25 or ≥25), KPS (100, 90, 80, or ≤70), blood pressure (pre-hypertensive or hypertensive), payer status (Medicare, private, or other), practice region (Midwest, Northwest, South, or West), exposure to corticosteroids at the initiation of second-line treatment (yes or no), degree of resection (no biopsy, partial excision, or excision) and therapy (non-bevacizumab, bevacizumab monotherapy or bevacizumab combination) received in the second-line setting.

Propensity score-adjusted sensitivity analyses were conducted to evaluate the relationship between treatment choice and subsequent survival. These analyses are particularly useful for studies with small sample sizes, and adjust for preexisting group differences that may lead to differences in treatment. Cox regression models, stratified on the quintiles of the propensity score, were constructed. These evaluations were conducted in all patients, and the scores determined for each patient were used as an adjustment variable in the Cox proportional hazard regression.

The median number of treatment cycles and duration of treatment, with interquartile ranges (IQR), were calculated for bevacizumab-containing regimens. Patients who received only one dose of treatment or who remained on treatment at the end of follow-up were excluded from these analyses.

All statistical analyses were conducted using SAS^®^ 9.2 (SAS Institute, Inc., Cary, NC).

## Results

### Study population characteristics

**Fig. 1:**
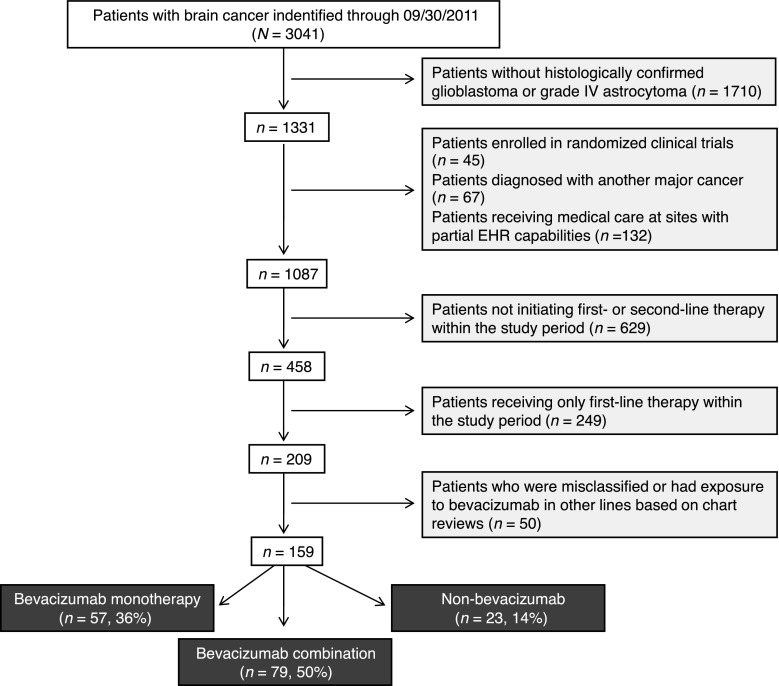
Patient selection procedure. *EHR* electronic health record, *IV* intravenous

In the iKM database, the care of 3041 patients with brain cancer was documented between July 1, 2006, and June 30, 2011. Of these, a total of 209 patients were initially identified as having histologically confirmed glioblastoma and no diagnosis of another major cancer; had initiated second-line therapy for glioblastoma within the study period, and were not participants in a randomized clinical trial within the network. After chart review, patients were excluded because of participation in a randomized clinical trial outside of the network (n = 27), where they had received bevacizumab as adjuvant or as first-line therapy (*n* = 20), or had received second-line bevacizumab beyond the study period (*n* = 3). Consequently, 159 patients were analyzed in this study (bevacizumab monotherapy [*n* = 57]; bevacizumab combination [*n* = 79]; non-bevacizumab [*n* = 23]) (Fig. 1).

There were 249 patients meeting the other inclusion criteria that received first line therapy in the study period but did not receive second-line therapy. Of these, 189 were alive, 26 died prior to initiation of second-line therapy, and one was lost to follow-up. Vital status data were missing for 33 of the patients who only received first-line therapy in the study period.

### Patient demographics and treatment characteristics

Patient characteristics were similar in the treatment groups with regard to sex, BMI, hypertension status, and KPS at the start of second-line therapy (Table 1). Notably, patients in the bevacizumab-combination group had a lower median age at the start of second-line therapy (54 years) compared with those receiving bevacizumab monotherapy (61 years) or a non-bevacizumab regimen (58 years) (*P* = 0.0135). A relatively high proportion of patients in the bevacizumab-monotherapy group were treated in the South, whereas patients in the non-bevacizumab group tended to be treated in the West. Patients who received bevacizumab monotherapy or bevacizumab combination were more likely to have private insurance (60 %) than patients who received non-bevacizumab regimens (30 %).

**Table 1:** Patient and clinical characteristics at the time of second-line treatment by group

Characteristics,* n* (%)	All patients (*N* = 159)	Second-line treatment		
		Bevacizumab monotherapy (*n* = 57)	Bevacizumab combination (*n* = 79)	Non-bevacizumab (*n* = 23)
Median age, years (range)*	57 (19–82)	61 (30–77)	54 (24–82)	58 (19–78)
≥60 years	69 (43)	31 (54)	27 (34)	11 (48)
Male sex	90 (57)	30 (53)	45 (57)	15 (65)
BMI, median (range)	27.0 (17.0–46.8)	26.6 (18.1–46.8)	27.6 (17.0–41.8)	28.1 (21.6–39.2)
≥25.0	119 (75)	40 (70)	63 (80)	16 (70)
Practice region				
Midwest	19 (12)	8 (14)	11 (14)	0 (0)
Northeast	30 (19)	8 (14)	18 (23)	4 (17)
South	53 (33)	27 (47)	18 (23)	8 (35)
West	57 (36)	14 (25)	32 (41)	11 (48)
Payer status*				
Medicare	35 (22)	15 (26)	11 (14)	9 (39)
Private	88 (55)	33 (58)	48 (61)	7 (30)
Other	36 (23)	9 (16)	20 (26)	7 (30)
Blood pressure				
Normal^a^	58 (36)	22 (39)	27 (34)	9 (39)
Prehypertension^b^	85 (53)	26 (46)	45 (57)	14 (61)
Hypertension (I and II)^c^	14 (9)	9 (16)	5 (6)	0 (0)
Missing	2 (1)	0 (0)	2 (3)	0 (0)
KPS				
100	8 (5)	2 (4)	5 (6)	1 (4)
90	34 (21)	11 (19)	17 (22)	6 (26)
80	35 (22)	10 (17)	22 (28)	3 (13)
≤70	48 (30)	20 (34)	19 (24)	9 (39)
Missing	34 (21)	14 (24)	16 (21)	4 (17)
Median Follow-up Time (months)*	8.41	6.76	10.24	5.19
Prior surgery^d^	145 (91)	53 (91)	69 (88)	23 (100)
Median time since surgery, months (range)^d^	11 (2–124)	9 (3–51)	12 (3–124)	16 (2–52)
Cortiosteroiduse^d,e^	134 (84)	48 (83)	65 (83)	21 (91)
Prior radiation therapy	155 (97)	56 (98)	77 (97)	22 (96)
Second-line therapy (other than bevacizumab)				
Irinotecan	63 (40)	–	60 (76)	3 (13)
Carboplatin + irinotecan	8 (5)	–	8 (10)	–
Carboplatin + etoposide	1 (1)	–	–	1 (4)
Carboplatin	9 (6)	–	7 (9)	2 (9)
Etoposide	1 (1)	–	–	1(1)
Temozolomide	14 (9)	–	2 (3)	12 (52)
Carmustine	1 (1)	–	1 (1)	–
Lomustine-containing regimen	4 (3)	–	–	4 (17)
Sorafenib	1 (1)	–	1 (1)	–
Excision				
Biopsy and partial	92 (58)	34 (60)	44 (56)	14 (61)
Complete excision	62 (39)	20 (35)	33 (42)	9 (39)
Missing	5 (3)	3 (5)	2 (2)	0 (0)

*BM,* body mass index, *KPS* Karnofsky performance status

* Statistically significant at *P* < 0.05

^a^Systolic reading <120 mm Hg and diastolic reading <80 mm Hg

^b^Systolic reading of 120–139 mm Hg or diastolic reading of 80–89 mm Hg

^c^Hypertension I: Systolic reading of 140–159 mm Hg or diastolic reading of 90–99 mm Hg; hypertension II: systolic reading ≥160 mm Hg or diastolic reading ≥100 mm Hg

^d^Data were obtained from both iKM database and electronic chart review; other variables were extracted from iKM

^e^Collected at any time point of the study period

**Table 2:** Adverse events of any grade related to second-line treatment by group

Adverse event, *n* (%)^**a**^	All patients (*N* = 159)	Second-line treatment		
		Bevacizumab monotherapy (*n* = 57)	Bevacizumab combination (*n* = 79)	Non-bevacizumab (*n* = 23)
Treatment-related hypertension	13 (8)	5 (9)	7 (9)	1 (4)
Hemorrhage/bleeding	2 (1)	2 (4)	–	–
Cerebral hemorrhage	2 (1)	2 (4)	–	–
Other hemorrhage	–	–	–	–
Gastrointestinal perforation	4 (3)	–	3 (4)	1 (4)
Thromboembolic events^b^	12 (8)	6 (11)	5 (6)	1 (4)
Proteinuria	9 (6)	2 (4)	7 (9)	–
Wound-healing complications^c^	2 (1)	1 (2)	1 (1)	–
Abscesses and fistulae	1 (1)	–	1 (1)	–

^a^Multiple adverse events were possible in a single patient

^b^Including venous and arterial thromboembolic events

^c^Including infections associated with postsurgical wounds

The majority of patients in each group had received surgery (inclusive of biopsy, complete resection or partial resection) and radiation in an earlier treatment setting (see Table 1). Overall median time since surgery, indicative of time to first progression, was 11 months (range 2–124) for all patients, with no significant differences observed between groups. The use of corticosteroids at the time of starting second-line treatment was consistent across the treatment groups.

The composition of second-line therapy varied between the bevacizumab-combination and non-bevacizumab groups (see Table 1). Patients who were treated with bevacizumab and another agent most commonly received irinotecan with or without carboplatin (68/79). In contrast, temozolomide was the preferential second-line chemotherapy used for patients treated with non-bevacizumab regimens (12/23), followed by lomustine-containing regimens (4/23) and single-agent irinotecan (3/24). Treatment sequence analysis revealed that an additional 3 patients in the non-bevacizumab group were treated with temozolomide as first line or adjuvant therapy.

In the bevacizumab-containing groups, the median number of treatment cycles (6 [IQR: 4–12] vs. 8 [IQR: 4–14]) and the duration of treatment (98 days [IQR: 56–155] vs. 154 days [IQR: 71–269]) were shorter in the bevacizumab-monotherapy group than in the bevacizumab-combination group, respectively, although the interquartile ranges were overlapping.

### Effectiveness outcomes

**Fig. 2:**
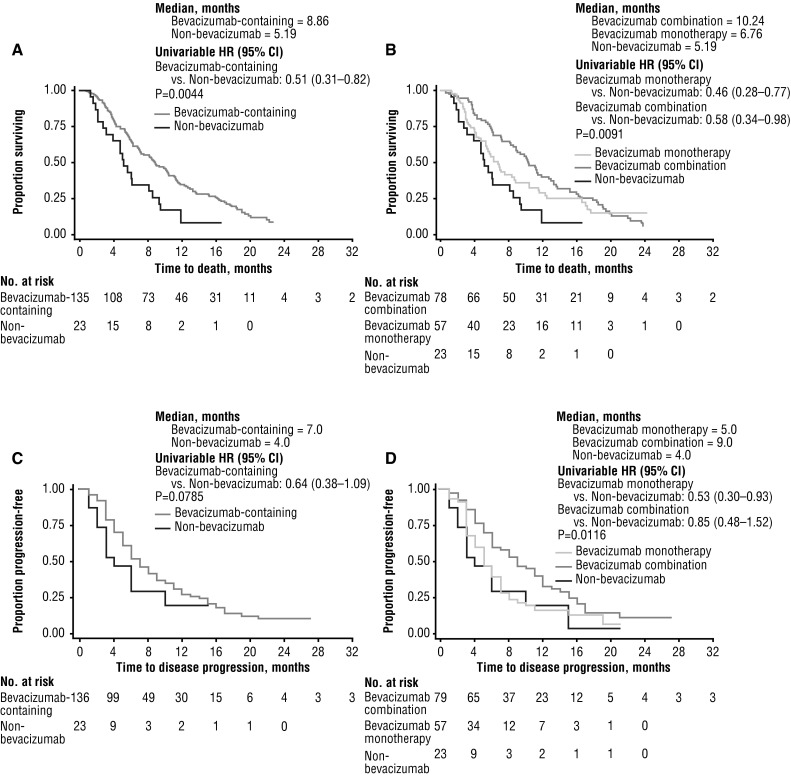
Kaplan-Meier estimates of OS and PFS for patients with recurrent glioblastoma: **a** OS for patients receiving second-line bevacizumab-containing therapy or non-bevacizumab therapy and **b** OS for patients receiving second-line bevacizumab monotherapy, bevacizumab-combination, or non-bevacizumab therapy. **c** PFS for patients receiving second-line bevacizumab-containing therapy or non-bevacizumab therapy and **d** second-line bevacizumab monotherapy, bevacizumab-combination, or non-bevacizumab therapy. *CI* confidence interval, *HR* hazard ratio, *OS* overall survival, *PFS* progression-free survival

At the end of follow-up, 141 (89 %) patients had died, 17 (11 %) were lost to follow-up, and information was unavailable for 1 patient (0.6 %). In the overall population, the median OS from the beginning of second-line therapy was 8.41 months (95 % CI, 6.27–9.86) by unadjusted analyses; the bevacizumab monotherapy, bevacizumab combination, and non-bevacizumab groups had unadjusted median survival of 6.76, 10.24, and 5.19 months, respectively. The Kaplan–Meier estimate for OS was significantly longer in patients who received second-line bevacizumab (monotherapy or combination) (8.86 months; 95 % CI 7.06–10.44) compared with patients in the non-bevacizumab group (5.19 months; 95 % CI 3.12–8.11) (log-rank test *P* = 0.0044) (Fig. 2a). When evaluating all three treatment cohorts, OS was increased in the bevacizumab-combination group relative to both the bevacizumab-monotherapy and the non-bevacizumab groups (log-rank test, *P* = 0.0091) (Fig. 2b).

The estimated median PFS in all patients treated with bevacizumab (7.00 months; 95 % CI 6.00–9.00) was longer than in those receiving a second-line regimen not containing bevacizumab (4.00 months; 95 % CI 2.00–10.00), but this did not reach statistical significance (log-rank test; *P* = 0.0785) (Fig. 2c). The 6-month PFS rates in the combined bevacizumab groups and the non-bevacizumab group were 51.39 % (95 % CI 42.25–59.80) and 29.05 % (95 % CI 10.99–50.06), respectively. In the unadjusted analysis, the median PFS in the bevacizumab-combination group was 9.00 months (95 % CI 6.00–12.00), and was significantly longer than that reported in the other two cohorts (log-rank test, *P* = 0.0116) (Fig. 2d).

After adjusting for confounding variables, the multivariable Cox model demonstrated that the use of second-line bevacizumab was associated with significantly improved OS (hazard ratio [HR] 0.45; 95 % CI 0.26–0.77), relative to the use of non-bevacizumab regimens as second-line treatment, while improvements in PFS (HR 0.69; 95 % CI 0.37–1.28), were not statistically significant. Moreover, both bevacizumab monotherapy and bevacizumab-combination therapy trended toward superior OS (HR 0.56 [95 % CI 0.31–1.03] and HR 0.34 [95 % CI 0.21–0.68], respectively; *P* = 0.0039) and PFS (HR 0.98 [95 % CI 0.50–1.92] and HR 0.52 [95 % CI 0.27–1.01 respectively; *P* = 0.0174) when compared with non-bevacizumab therapy (Tables 3, 4). In Cox models adjusted by propensity scores, similar improvements in HRs for survival outcomes were observed for both the bevacizumab monotherapy and bevacizumab-combination therapy with respect to OS, and for the bevacizumab-combination therapy with respect to PFS when compared to non-bevacizumab therapy (data not shown).

**Table 3:** Cox proportional hazards analysis of OS

Category	Reference (n)	Variable (n)	HR (95 % CI)
Treatment cohort	Non-bevacizumab (n = 23)	Bevacizumab monotherapy (n = 58)	0.56 (0.31–1.03)
		Bevacizumab combination (n = 78)	0.34 (0.21–0.68)
Age	<60 years (n = 90)	≥60 years (n = 69)	0.71 (0.45–1.12)
Sex	Female (n = 69)	Male (n = 90)	0.99 (0.66–1.51)
BMI	<25 (n = 40)	≥25 (n = 119)	1.09 (0.69–1.74)
Region	Midwest (n = 19)	Northwest (n = 30)	0.99 (0.49–1.99)
		South (n = 53)	0.82 (0.42–1.58)
		West (n = 57)	1.14 (0.59–2.19)
Baseline KPS	100 (n = 7)	90 (n = 33)	1.15 (0.43–3.04)
		80 (n = 33)	1.10 (0.42–2.90)
		≤70 (n = 45)	0.75 (0.28–1.99)
		Missing (n = 41)	1.15 (0.43–3.03)
Baseline BP	Normal (n = 58)	Pre-hypertension (n = 85)	0.91 (0.59–1.39)
		Hypertension (n = 14)	0.94 (0.45–1.96)
Payor status	Medcare (n = 35)	Private (n = 88)	0.93 (0.53–1.65)
		Other (n = 22)	1.35 (0.70–2.60)
Baseline steroid	No (n = 14)	Yes (n = 126)	0.75 (0.37–1.49)
Excision	Biopsy and partial (n = 92)	Complete excision (n = 62)	1.22 (0.83–1.81)

**Table 4:** Cox proportional hazards analysis of PFS

Category	Reference (n)	Variable (n)	HR (95 % CI)
Treatment cohort	Non-bevacizumab (n = 23)	Bevacizumab monotherapy (n = 58)	0.98 (0.50–1.92)
		Bevacizumab combination (n = 78)	0.52 (0.27–1.01)
Age	<60 years (n = 90)	≥60 years (n = 69)	1.03 (0.63–1.67)
Sex	Female (n = 69)	Male (n = 90)	1.21 (0.79–1.87)
BMI	<25 (n = 40)	≥25 (n = 119)	0.94 (0.57–1.53)
Region	Midwest (n = 19)	Northwest (n = 30)	1.34 (0.65–2.77)
		South (n = 53)	0.73 (0.37–1.43)
		West (n = 57)	0.98 (0.49–1.96)
Baseline KPS	100 (n = 7)	90 (n = 33)	1.91 (0.52–6.99)
		80 (n = 33)	1.17 (0.31–4.40)
		≤70 (n = 45)	1.38 (0.37–5.11)
		Missing (n = 41)	1.39 (0.37–5.15)
Baseline BP	Normal (n = 58)	Pre-hypertension (n = 85)	0.77 (0.49–1.19)
		Hypertension (n = 14)	1.01 (0.47–2.16)
Payor status	Medcare (n = 35)	Private (n = 88)	1.52 (0.83–2.78)
		Other (n = 22)	1.57 (0.75–3.29)
Baseline steroid	No (n = 14)	Yes (n = 126)	0.65 (0.31–1.33)
Excision	Biopsy and partial (n = 92)	Complete excision (n = 62)	1.11 (0.72–1.71)

Multivariable analyses also showed that patients receiving bevacizumab combination had a trend toward longer OS (HR 0.34; 95 % CI 0.21–0.68) and longer PFS (HR 0.52; 95 % CI 0.27–1.01) than those receiving bevacizumab monotherapy. The Cox models, which included a propensity score adjustment, indicated a HR for OS of 0.38 (95 % CI 0.25–0.58) and PFS of 0.61 (95 % CI 0.30–1.23) in favor of bevacizumab-combination therapy. No additional factors (i.e., categories according to age, sex, BMI, KPS, blood pressure status, payer status, practice region, exposure to corticosteroids, or degree of resection) were associated with improved survival outcomes (Tables 3, 4).

Only 29 patients (18 %) received third-line therapy; 13 patients in the second-line bevacizumab monotherapy group, 9 patients in the second-line non-bevacizumab group, and 7 patients in the bevacizumab combination group received third-line therapy, respectively. A total of 107 patients (67 %) died prior to receiving third-line therapy, 16 patients (10 %) were still receiving second-line therapy at the end of the study period, and 7 patients (4 %) had no additional data regarding the use of third-line therapy.

### Safety

In general, there was a low incidence of bevacizumab-associated adverse events in the bevacizumab-monotherapy and bevacizumab-combination groups (Table 2). Venous and arterial thrombosis (11 %) and treatment-related hypertension (9 %) were adverse events reported most commonly among patients receiving bevacizumab monotherapy. In the bevacizumab-combination group, treatment-related hypertension (9 %) and proteinuria (9 %) were observed most frequently. Only three patients in the second-line non-bevacizumab group reported treatment-related adverse events (one case each of treatment-related hypertension, gastrointestinal perforation, and thromboembolism).

At the start of second-line therapy, the percentage of patients on corticosteroids was similar across the treatment groups (78–83 %). Corticosteroid use decreased during second-line treatment, with similar reductions in the number of patients requiring corticosteroids across all groups (Table S1).

## Discussion

This novel analysis of EHR data in a large community-practice setting allowed for the investigation of clinical outcomes in patients treated with second-line bevacizumab monotherapy, bevacizumab-combination regimens, and non-bevacizumab–containing treatment for recurrent glioblastoma. The analysis revealed that the use of bevacizumab-containing regimens, when compared separately or together, was associated with significantly improved survival relative to the use of regimens that did not include bevacizumab. In the multivariable analysis that combined all patients receiving bevacizumab, the HRs for OS and PFS were 0.45 and 0.69, respectively. These data are consistent with reports from phase 2 trial analyses in progressive glioblastoma, including the pivotal BRAIN study [9]. In addition, the median OS and PFS values (8.86 and 7.00 months, respectively) in the combined bevacizumab group are similar to outcomes reported in a meta-analysis of bevacizumab treatment for recurrent glioblastoma (median OS and time to progression of 9.3 and 6.1 months, respectively) [25] as well as those reported in retrospective studies (range of median OS values: 8.5–11.5 months; range of median PFS values: 4.3–7.6 months) [18–20, 22]. This concordance provides some assurance that the data from the current analysis reflect clinical practice.

Subgroup analyses also revealed a trend toward both longer OS (HR 0.38; 95 % CI 0.25–0.58) and PFS (HR 0.61; 95 % CI 0.30–1.23) for patients treated with bevacizumab combination than those treated with bevacizumab monotherapy. To our knowledge, no previous studies have indicated a benefit with the addition of chemotherapy to bevacizumab in recurrent glioblastoma. In the BRAIN study, Friedman and colleagues reported that 95 % CIs for median OS and PFS were largely overlapping between the bevacizumab monotherapy and bevacizumab-irinotecan arms, although the study was neither designed nor powered to compare treatment arms [9].

Importantly, the multivariable analyses in the present analysis controlled for a number of patient and disease characteristics. While the median age in the bevacizumab-combination group was younger than in the bevacizumab-monotherapy group, age was not significantly associated with either OS or PFS in this analysis. There did, however, appear to be differences in the duration of treatment in the two bevacizumab groups, which may have impacted outcomes.

The current analysis found that clinical factors, including age, sex, KPS, and degree of resection at the start of second-line treatment, were not prognostic for survival. While these findings are congruous with those from a meta-analysis of phase 2 bevacizumab salvage trials for recurrent glioblastoma [26], pooled analyses from a number of research groups have shown significant associations between age, performance status, and/or corticosteroid use on survival in recurrent glioma trials [27–29].

Bevacizumab treatment appeared to be well tolerated, and the incidence of bevacizumab-related adverse events was similar to that observed in previous studies in recurrent glioblastoma [9–11]. The incidence of intracranial hemorrhage was low, with only two reported cases (4 %) in the bevacizumab-monotherapy group.

A number of limitations are inherent in the study design. EHR data are not collected for comparative research purposes but for clinical practice reasons, and variations in data-collection methods and the reporting practices of individual physicians may exist [30]. To circumvent potential misclassification errors, electronic chart reviews were conducted to validate EHR data for critical parameters and information from the SSDI was used to supplement vital status data.

Other limitations include the lack of integration of proposed Response Assessment in Neuro-Oncology criteria [31], heterogeneity of treatments in the bevacizumab-combination and non-bevacizumab groups, the possibility of unobserved selection bias that cannot be accounted for by statistical adjustment, the retrospective observational nature of the study, and the small size of the patient population receiving non-bevacizumab treatment. The authors recognize that a relatively small number of patients received third-line therapy (approximately 18 % of all patients in the study cohort). This is lower than is observed in prospective clinical trials, and is explained by the retrospective nature of the study in which the majority of patients died before receiving third-line therapy and others were still receiving second-line therapy at the end of the study period. Additionally, the presence of radiation necrosis was not documented among patients receiving second line therapy in this study, therefore bias resulting from this potential therapeutic benefit of bevacizumab cannot be excluded from the analysis [32]. Treatment patterns supported within the McKesson evidence-based guidelines may also differ to some degree from treatment patterns in other community-based practices, as well as from sites not utilizing the full EHR capabilities. As this was not a prospective study, it is possible that selection bias may have influenced the results, since treating physicians may have based treatment decisions on patient characteristics such as overall fitness and/or comorbidities.

Lastly, prognostic biomarker data were not available for our study patients, which may introduce important biological imbalances into the study arms, as several prognostic molecular markers have previously been identified and efforts continue to identify markers with predictive value [33–35].

## Conclusions

In this retrospective study of patients with recurrent glioblastoma, bevacizumab-containing regimens were associated with significant improvements in OS and PFS relative to non-bevacizumab regimens by unadjusted and multivariable analyses. Despite the limitations inherent in the study design, the use of a large, geographically dispersed cohort of community-based patients, confirmatory propensity score adjusted analyses, and consistency with previously reported findings, further supports the clinical value and acceptable safety profile of bevacizumab treatment for progressive, bevacizumab-naive glioblastoma in a real-world setting. Recently completed phase 3 trials investigated the incorporation of bevacizumab into frontline regimens for newly diagnosed disease [36, 37] and did not demonstrate an improvement in overall survival. However, this retrospective study complements previous prospective results supporting the clinical value of bevacizumab in the treatment of recurrent glioblastoma.

## Electronic supplementary material

Below is the link to the electronic supplementary material.

Supplementary material 1 (DOCX 13 kb)
