# Supplementary material for: Clinical outcomes with bevacizumab-containing and non-bevacizumab–containing regimens in patients with recurrent glioblastoma from US community practices
Source: J Neurooncol. 2015 Mar 15;122(3):595–605. doi: 10.1007/s11060-015-1752-y (PMC4436682; doi:10.1007/s11060-015-1752-y)
Supplement: Supplementary file 1 — Supplementary material 1 (DOCX 13 kb) [file 11060_2015_1752_MOESM1_ESM.docx]

**Supplementary Table** Use of corticosteroids during second-line treatment for glioblastoma by group

| **Corticosteroid use, *n* (%)** | **All patients**  **(*N* = 159)** | **Second-line treatment** | | | ***P* value^a^** |
| --- | --- | --- | --- | --- | --- |
|  |  | **Bevacizumab monotherapy**  **(*n* = 57)** | **Bevacizumab combination**  **(*n* = 79)** | **Non-bevacizumab**  **(*n* = 23)** |  |
| Baseline | 126 (79) | 45 (79) | 62 (78) | 19 (83) | 0.9097 |
| 2 months | 53 (33) | 23 (40) | 19 (24) | 11 (48) | 0.0388 |
| 3 months | 44 (28) | 19 (33) | 18 (23) | 7 (30) | 0.3785 |
| 4 months | 44 (28) | 20 (35) | 19 (24) | 5 (22) | 0.2882 |
| 5 months | 35 (22) | 17 (30) | 14 (18) | 4 (17) | 0.2060 |
| 6 months | 38 (24) | 18 (32) | 16 (20) | 4 (17) | 0.2275 |

^a^Chi-squared test
